# A Prospective Study to Examine Responsiveness and Minimally Important Differences (MIDs) for the CLEFT-Q Scales Following Three Cleft-Specific Operations

**DOI:** 10.1177/10556656211064479

**Published:** 2021-12-14

**Authors:** A Miroshnychenko, C Rae, K Wong Riff, CR Forrest, T Goodacre, MC Swan, R Slator, J Goldstein, A Thoma, K Harman, A Klassen

**Affiliations:** 1 Department of Health Research Methods, Evidence and Impact, 3710 McMaster University, Hamilton, Canada; 2 Department of Health Research Methods, Evidence and Impact, and Pediatrics, 3710 McMaster University, Hamilton, Canada; 3 Division of Plastic and Reconstructive Surgery, The Hospital for Sick Children, Toronto, Canada; 4 Department of Plastic & Reconstructive Surgery, Oxford University Hospitals NHS Foundation Trust, England, UK; 5 Department of Plastic & Reconstructive Surgery, Oxford University Hospitals NHS Foundation Trust, Spires Cleft Centre, 11269 John Radcliffe Hospital, England, UK; 6 Department of Plastic and Reconstructive Surgery, Birmingham Children's Hospital NHS Foundation Trust, England, UK; 7 Department of Plastic Surgery, 6595 University of Pittsburgh, PA (Pitt), PA, USA; 8 Division of Plastic Surgery, Department of Surgery, and Department of Health Research Methods, Evidence and Impact, 3710 McMaster University, Hamilton, Canada; 9 Department of Pediatrics, 3710 McMaster University, Hamilton, Canada

**Keywords:** bone grafting, orthognathic surgery, rhinoplasty, psychological assessment, quality of life

## Abstract

**Objective:**

The aim of this study was to examine internal responsiveness and estimate minimally important differences (MIDs) for CLEFT-Q scales.

**Design:**

In this prospective cohort study, participants completed the CLEFT-Q appearance and health-related quality of life (HRQL) scales before and six months after cleft-related surgery.

**Setting:**

Seven cleft centres in Canada, USA and UK participated.

**Patients/Participants:**

Patients were ages 8–29 years with CL/P.

**Interventions:**

Patients underwent rhinoplasty, orthognathic or cleft lip scar revision surgery.

**Main Outcome Measure(s):**

Internal responsiveness was examined using Cohen's d effect sizes (ESs) based on the following interpretation: 0.20–0.49 small, 0.50–0.79 moderate and ≥ 0.80 large. MIDs were estimated using two distribution-based approaches.

**Results:**

Participants had a rhinoplasty (n = 31), orthognathic (n = 21) or cleft lip scar revision (n = 18) surgery. Most participants were males (56%) and aged 8–11 years (41%). Following rhinoplasty, ESs were larger for the nose (0.92, p = 0.001) and nostrils (0.94, p < 0.001) scales than for the face scale (0.51, p = 0.003). MIDs ranged between 6.2–10.4. For orthognathic surgery, larger ES was observed for the jaws scale (1.80, p < 0.001) compared with the teeth (1.16, p < 0.001), face (1.15, p = 0.001) and lips (0.94, p < 0.001) scales. MIDs ranged between 5.9–14.4. In the cleft lip scar revision sample, the largest ES was observed for the nose scale (0.76, p = 0.03), followed by lips (0.58, p = 0.009) and cleft lip scar (0.50, p = 0.043) scales. MIDs ranged between 6.4–12.3.

**Conclusions:**

CLEFT-Q detected change in key outcomes for three cleft-specific surgeries, providing evidence of its responsiveness. Estimated MIDs will aid in interpreting this PROM.

## Introduction

The CLEFT-Q is a patient reported outcome measure (PROM) for children and young adults with a cleft lip and/or palate (CL/P). PROMs are tools designed to collect reports that come directly from patients about how they function or feel in relation to a health condition and its therapy, without interpretation by a physician or anyone else (Valderas et al., 2008). These tools must be carefully designed and tested to ensure their validity and reliability. Measurement instruments that are not valid, reliable, and responsive to change cannot accurately examine patients’ experiences in regard to their medical condition and its treatment.

The development of the CLEFT-Q scales was a multidisciplinary and multisite initiative. Collaboration with international teams ensured that the rigorous development and validation processes of the CLEFT-Q scales accounted for multicultural perspectives on cleft-related care. The rapid uptake of CLEFT-Q scales in 45 countries, and its translation into 22 languages to date is evidence of its useful, comprehensive and relevant nature. The selection of CLEFT-Q for inclusion in the ICHOM (International Consortium for Health Outcome Measurement) cleft standard set provides a means for hospitals worldwide to use the scales in clinical care and research, with potential for global benchmarking (Allori et al., 2017). The CLEFT-Q scales were developed via three phases using a modern psychometric approach, i.e., Rasch measurement theory (Wong Riff et al., 2017). In the first phase, 138 patients with CL/P from six countries were interviewed, and data were used to form 13 scales that measure appearance, facial function and health-related quality of life (HRQL) (Tsangaris et al., 2017). In the second phase, scales were field-tested internationally with 2434 patients to examine reliability and validity, and to develop a common scoring algorithm for each scale for international use (Klassen et al., 2018). The third phase, the focus of this publication, investigated responsiveness of the CLEFT-Q scales and determined minimally important differences (MIDs) to aid in interpretation of the scale scores.

Responsiveness is the ability of an instrument to detect statistically significant and clinically important changes over time (Guyatt et al., 1989). Responsiveness can be internal and external (Guyatt et al., 1989). Internal responsiveness, examined in this study, is the ability of a measurement tool to detect statistically significant and clinically important changes over time (Husted et al., 2000; Chiu et al., 2018). External responsiveness is the extent to which change in scores for a new instrument relate to change in scores on another instrument that examines the condition of interest (Husted et al., 2000). Establishing responsiveness of the CLEFT-Q scales is an essential component of its psychometric assessment, as this tool is an evaluative measure of appearance, function and HRQL for patients with CL/P.

In addition to responsiveness, it is important to define the thresholds for clinically important changes, which aid in the interpretability of an instrument. The minimal clinically important difference or MID is the smallest difference in scores measuring the outcome of interest that patients perceive as an important deterioration or improvement (Jaeschke et al., 1989; Schünemann et al., 2005; Schünemann and Guyatt, 2005). MIDs have been reported to be useful in terms of the following: 1) prioritizing the patients’ perspective, 2) informing judgements about the success of an intervention, and 3) helping to estimate the sample size for clinical trials. A disadvantage of MIDs is that estimates are known to vary across patients and patient groups and therefore should be applied only to the patient population for which the MID estimates were calculated (Santanello et al., 1999; Schünemann et al., 2005; Schünemann and Guyatt, 2005).

In addition to enabling healthcare professionals to interpret changes in scale scores, and providing a basis for estimating sample sizes in prospective randomized controlled trials, the use of MIDs to identify the most effective treatments in comparative studies may help to reduce variability in treatment protocols within and between countries, such as that detected in Americleft, Eurocleft and Scandicleft studies (Deyo et al., 1991; Guyatt et al., 2002; Locker et al., 2004; Semb et al., 2005; Long et al., 2011; Semb et al., 2017).

The first objective of this study was to assess the internal responsiveness of the CLEFT-Q scales by examining whether change scores collected before and six months after three cleft-related surgeries (rhinoplasty, orthognathic and cleft lip scar revision) were statistically significant, and in accordance with prespecified hypotheses. The second objective was to determine the clinically important change or MIDs for each CLEFT-Q scale for three cleft-related operations.

## Methods

### Data Collection

#### The CLEFT-Q

The CLEFT-Q is a condition-specific PROM for children and young adults with CL/P. It is composed of 12 independently functioning scales and one checklist. The scale scores are calculated by converting the raw scores into Rasch transformed scores ranging from 0 to 100. Higher scores reflect a better outcome.

This study was conducted between January 2018 and October 2019 in three countries (ie, Canada, United States of America [USA] and United Kingdom [UK]) at seven cleft centers (ie, The Hospital for Sick Children, Children's Hospital of Pittsburgh, Queen Elizabeth Hospital Birmingham, Birmingham Women's and Children's Hospital, Great Ormond Street Hospital for Children, Broomfield Hospital and Oxford and Salisbury Cleft Centers). Research ethics approval was obtained at the sites in Canada and the USA and from the National Health Services and hospital sites in the UK.

Patients were eligible if they were ages eight to 29 years at the preoperative assessment for a rhinoplasty, orthognathic or cleft lip scar revision surgery. Individuals with a cognitive delay were excluded if they were unable to complete the questionnaire independently. The presence of cognitive delay was assessed by a mental health professional, and this information was retrieved from the participant's hospital chart. Each participant completed a core set of CLEFT-Q scales that measured appearance (ie, face, nose, nostrils) and HRQL (ie, psychological, social and school). Participants completed the scales before and six months after their cleft-related surgery. Additional appearance scales were administered to individuals undergoing the orthognathic and cleft lip scar revision operations (See Table 1). Data for the school scale, which was only completed by participants aged eight to 18 years, were excluded from the analyses.

**Table 1.** The CLEFT-Q Scales Included in the Assessment of Rhinoplasty, Orthognathic and Cleft Lip Scar Revision Surgeries.

Scale (# of items)	Rhinoplasty	Orthognathic	Cleft lip scar revision
Face* (9)	Y	Y	Y
Nose* (12)	Y	Y	Y
Nostrils* (6)	Y	Y	Y
Teeth (8)		Y	
Jaws (7)		Y	
Lips (9)		Y	Y
Cleft lip scar (7)			Y
Psychological* (10)	Y	Y	Y
Social* (10)	Y	Y	Y

*Core scales.

Recruitment methods at each site were based on their preferences and logistics (see Supplemental Appendix 1). Individuals who did not complete the postoperative assessment at the initial request (non-respondents) were contacted up to three times by phone or email. If no response was received after the third contact, the non-respondent was considered lost to follow up.

Data were entered into a REDCap database hosted at the coordinating site at McMaster University, Canada (Harris et al., 2009; Harris et al., 2019). Data were downloaded from REDCap into IBM SPSS Statistics for Mac, Version 26.0 (IBM Corporation, Armonk NY, USA for Windows®/Apple Mac®) for analyses.

### Analysis

The pre- and postoperative assessment scores for CLEFT-Q scales were calculated by converting the raw scores into Rasch transformed scores ranging from 0 to 100. The assumption of normality was examined through assessment of skewness and kurtosis and normality plots with significance tests. If the skewness and kurtosis values were below an absolute value of two, the data were considered normally distributed (Kim, 2013). If normality assumption was not met, the non-parametric equivalent test was used for the analysis.

In accordance with the Consensus-based Standards for the selection of health Measurement Instruments (COSMIN) recommendations for examining responsiveness, analyses were conducted separately for the three surgical procedures, as each procedure affects some facial features more than others (Mokkink et al., 2018). Paired sample t-tests were conducted to determine whether there was a statistically significant difference between the pre- and postoperative CLEFT-Q scores for each independently functioning scale included in the assessment of each surgical procedure. As such, each CLEFT-Q scale score was tested only once, eliminating the need for the Bonferroni correction of the p-value. The magnitude and direction of change was determined using two effect size (ES) estimators: 1) Cohen's d and 2) Standardized Response Mean (SRM). Cohen's d ESs were interpreted using the Cohen's criteria: 0.20–0.49 small, 0.50–0.79 moderate and ≥ 0.80 large (Cohen, 1988; Masood et al., 2014). Formulas used to calculate the distribution-based statistics are listed in Supplemental Appendix 2.

Two internal responsiveness hypotheses were tested. 1) the ESs on the appearance scales would be larger than on the HRQL scales, because the HRQL construct represents a more distal construct than appearance in the context of cleft-related surgery outcomes. For this hypothesis, the score changes on the HRQL scales were not expected to be as large as on the appearance scales. Further, the change to appearance is likely to occur before the change in HRQL, because surgery is considered to have an immediate impact on appearance. 2) the ESs on the appearance scales examining aspects of appearance most affected by surgery would be larger than on the appearance scales examining aspects of appearance less affected by surgery. The second hypothesis was based on the reasoning that the larger the impact of the surgery on a particular facial area, the more noticeable the visible change in that area would be, hence the larger the change on the appearance scale measuring that facial area.

The clinically important change (ie, MID) was calculated with two distribution-based approaches by calculating scale score change that pertains to the following: 1) ½ ES and 2) ½ SD of the preoperative mean scores for each scale following each cleft-related surgery.

## Results

### Descriptive Data

Sample characteristics are shown in Table 2. A total of 93 participants were included in the phase III study; 23 of these participants did not complete the postoperative assessment. The non-respondents to the postoperative follow up assessment were similar to respondents in terms of mean age (χ^2^ = 4.86, p = 0.18), type of operation (χ^2^ = 4.89, p = 0.18) and gender (χ^2^ = 4.22, p = 0.06), but were more likely to live in the UK rather than Canada or the USA (χ^2^ = 6.45, p = 0.04). The sample size by type of surgery were as follows: rhinoplasty (n = 31), orthognathic (n = 21) and cleft lip scar revision (n = 18). The average time between the pre- and postoperative assessments ranged between five months for rhinoplasty to seven months for cleft lip scar revision.

**Table 2.** Characteristics of Participants in the CLEFT-Q Phase III Study.

Characteristic	No. of participants at baseline (%) n = 70	No. of non-respondents (%) n = 23
Country		
Canada	25 (36%)	9 (39%)
UK	22 (31%)	12 (52%)
USA	23 (33%)	2 (9%)
Age, years		
8–11	4 (6%)	0 (0%)
12–15	12 (17%)	5 (22%)
16–20	39 (56%)	8 (35%)
≥ 21	15 (21%)	10 (44%)
Gender		
Female	39 (56%)	16 (70%)
Male	31 (44%)	7 (30%)
Student		
Yes	49 (70%)	13 (57%)
No	21 (30%)	10 (44%)
Cleft type		
Cleft lip only	2 (3%)	5 (22%)
Cleft palate only	2 (3%)	0 (0%)
Cleft lip and palate	57 (83%)	15 (65%)
Cleft lip and alveolus	8 (12%)	3 (13%)
Syndrome or craniofacial anomaly		
Yes	3 (4%)	2 (9%)
No	65 (96%)	20 (91%)
Operation type		
Rhinoplasty	31 (44%)	7 (30%)
Orthognathic	21 (30%)	6 (26%)
Cleft lip scar	18 (26%)	10 (44%)

### Rhinoplasty

Statistically significant changes between the pre- and postoperative scores were observed for the following CLEFT-Q scales: face (mean difference = 7.6, SD = 19.0, p = 0.033), nose (mean difference = 17.1, SD = 26.0, p = 0.001) and nostrils (mean difference = 25.3, SD = 29.7, p < 0.001) (See Table 3). The difference between the pre- and postoperative scores for the CLEFT-Q HRQL scales were not significant. The magnitude of change was considered large on the nose and nostrils scales, and moderate on the face scale (See Table 4). Both hypotheses regarding the magnitude of change on the CLEFT-Q scales for the rhinoplasty operation were supported.

**Table 3.** CLEFT-Q Scale Results pre and Post-Operatively.

Scale	Preoperative Mean (SD)			Postoperative Mean (SD)		
	Rhinoplasty (n = 31)	Orthognathic (n = 21)	Cleft lip scar revision (n = 18)	Rhinoplasty (n = 31)	Orthognathic (n = 21)	Cleft lip scar revision (n = 18)
Face	49.4 (12.4)	42.6 (12.4)	48.9 (8.7)	57.0 (17.1)	60.9 (18.9)	56.4 (17.8)
Nose	49.8 (19.4)	35.4 (28.8)	45.1 (13.2)	66.9 (17.7)	47.1 (30.3)	57.3 (18.9)
Nostrils	33.0 (20.9)	33.4 (27.4)	33.3 (19.4)	58.3 (31.8)	44.9 (33.9)	47.7 (29.2)
Teeth	-	44.8 (11.7)	-	-	66.7 (23.9)	-
Jaws	-	35.7 (15.6)	-	-	72.8 (24.7)	-
Lips	-	39.8 (18.5)	39.3 (12.9)	-	62.1 (28.0)	54.8 (22.5)
Cleft lip scar	-	-	43.0 (24.7)	-	-	54.1 (19.3)
Psychological	68.9 (16.1)	64.0 (19.9)	64.9 (13.3)	71.3 (20.7)	69.7 (17.3)	64.9 (20.1)
Social	74.6 (13.3)	76.9 (17.4)	70.7 (15.8)	76.5 (18.8)	80.8 (20.4)	77.8 (17.5)

**Table 4.** Effect Sizes for Rhinoplasty, Orthognathic and Cleft lip Scar Revision Surgeries.

Scale	Cohen's d			Standardized Response Mean		
	Rhinoplasty	Orthognathic	Cleft lip scar revision	Rhinoplasty	Orthognathic	Cleft lip scar revision
Face	0.51	1.15	-	0.40	0.88	-
Nose	0.92	0.40	0.76	0.66	0.58	0.56
Nostrils	0.94	-	-	0.85	-	-
Teeth	-	1.16	-	-	1.14	-
Jaws	-	1.80	-	-	1.60	-
Lips	-	0.94	0.58	-	0.94	0.72
Cleft lip scar	-	-	0.50	-	-	0.55
Psychological	-	-	-	-	-	-
Social	-	-	**-**	-	-	-

Interpretation Note: Cohen's ES criteria: 0.20–0.49 small, 0.50–0.79 moderate and ≥ 0.80 large.

#### Orthognathic Surgery

Statistically significant changes were detected between the pre- and postoperative scores for the following CLEFT-Q scales: face (mean difference = 18.3, SD = 20.9, p = 0.001), nose (mean difference = 11.7, SD = 20.0, p = 0.017), teeth (mean difference = 21.9, SD = 19.2, p < 0.001), jaws (mean difference = 37.1, SD = 23.1, p < 0.001) and lips (mean difference = 22.3, SD = 23.7, p < 0.001) (See Table 3). The differences between the pre- and postoperative scores for either the nostrils or HRQL scales were not significant. The magnitude of change was classified as large for the face, teeth, lips and jaws scales, and small for the nose scale (See Table 4). Both the first and second hypotheses regarding the magnitude of change on the CLEFT-Q scales following the orthognathic operation were supported.

#### Cleft Lip Scar Revision

Statistically significant change between the pre- and postoperative scores was observed for the following CLEFT-Q scales: nose (mean difference = 12.2, SD = 22.1, p = 0.035), lips (mean difference = 15.5, SD = 21.5, p = 0.009) and cleft lip scar (mean difference = 11.1, SD = 20.0, p = 0.043) (See Table 3). The difference between pre- and postoperative scores were not observed for the face, nostrils and HRQL scales. Magnitude of change was moderate on the cleft lip scar, lips and nose scales (See Table 4).

The ESs on the appearance scales were larger than on the HRQL scales supporting the first hypothesis. The pattern of ESs for the cleft lip scar revision sample differed from the hypothesised, with the largest ESs on the nose scale, followed by the lips and the cleft lip scar scales.

### MIDs

MIDs for the CLEFT-Q scales included in the assessment of the rhinoplasty, orthognathic and cleft lip scar revision operations using the ½ ES and ½ SD approaches ranged between 5.9–14.4 (see Table 5).

**Table 5.** The Minimally Important Differences (MIDs) for the CLEFT-Q Scales Following Rhinoplasty, Orthognathic and Cleft Lip Scar Revision Surgeries.

Scale	MID ½ SD			MID ½ ES		
	Rhinoplasty	Orthognathic	Cleft lip scar revision	Rhinoplasty	Orthognathic	Cleft lip scar revision
Face	6.2	6.2	-	9.5	10.5	-
Nose	9.7	14.4	6.6	12.9	10.0	11.1
Nostrils	10.4	-	-	14.9	-	-
Teeth	-	5.9	-	-	9.6	-
Jaws	-	7.8	-	-	11.6	-
Lips	-	9.2	6.4	-	11.9	10.8
Cleft lip scar	-	-	12.3	-	-	10.0
Psychological	-	-	-	-	-	-
Social	-	-	-	-	-	-

## Discussion

We detected statistically significant improvements in the patients’ perspective of appearance of their face, nose, nostrils, jaws, lips and cleft lip scar on CLEFT-Q scales following three cleft-related surgeries. Five of six hypotheses about the magnitude of change were supported, which provided evidence of the responsiveness of the CLEFT-Q. These findings of responsiveness are in addition to their validity and reliability demonstrated in earlier studies (Tsangaris et al., 2017; Klassen et al., 2018). Furthermore, the estimated MIDs for scales relevant to rhinoplasty, orthognathic and cleft lip scar revision operations enhance the interpretability of the CLEFT-Q scales.

 The CLEFT-Q scales for the part of the face targeted by surgery demonstrated the greatest magnitude of change compared to scales for other areas of the face. Five of six (83%) hypotheses comparing the magnitude of change on one scale with another were supported. For appearance, these findings showed that CLEFT-Q scales were able to detect different amounts of change on various appearance scale depending on the type of treatment received. The only exception to expectations was the ES hypothesized for the cleft lip scar revision group, where the largest change was observed on the nose scale, rather than the lips and cleft lip scar scales. This result is not entirely surprising, as the cleft lip scar revision surgery has previously demonstrated improvement in appearance of the nose and nasolabial region (Mercado et al., 2014; Rothermel et al., 2020). Furthermore, lip revision scars tend to be more visible and take longer to mature than the rhinoplasty scars, thus offering another explanation for smaller improvement on the lips and cleft lip scar scales than on the nose scale within a six-month timeframe. Additionally, a small sample size (n = 18) may have contributed to this unexpected result.

MIDs were determined by surgery type for each of the CLEFT-Q scales to provide users with findings to guide interpretation of results. An exploratory approach via two distribution-based methods was used to estimate the MIDs. The MID estimates varied between methodological approaches and surgery types. For example, MIDs for the CLEFT-Q nose scale were 9.7 (½ SD) and 13.0 (½ ES approach) in the rhinoplasty sample, compared with 14.4 (½ SD) and 10.0 (½ ES) in the orthognathic sample. The variability we found in MIDs for the two approaches could be related to the relatively small sample sizes for each operation. Further efforts to determine MIDs in studies with larger samples will lead to more precise estimates. For now, the MIDs calculated with both approaches can be used to approximate whether a change detected by the scales is clinically significant.

The MIDs calculated in this study compare to the MIDs computed for other plastic surgery specific PROMs. For example, the MIDs estimated for the BREAST-Q Satisfaction with Breast scale one year after the surgery, based on data from 3052 patients, ranged between 3.2–5.1 for autologous reconstruction, 3.3–5.3 for alloplastic reconstruction and 3.3–5.6 for radiation therapy (Voineskos et al., 2020). Among the two studies, different distribution-based approaches (i.e, score changes corresponding to 1/2 SD vs 1/5 SD) were used to calculate the MIDs, which might account for the variability in the estimates. As the distribution-based approach relies solely on the statistical characteristics of the PROM scores rather than on the patients’ perspective, this comparison further highlights the need to compare MIDs calculated using the distribution-based approaches with MIDs calculated using the anchor-based approach (Bernstein and Mauger, 2016).

 Although the CLEFT-Q appearance scales were responsive to change following cleft-specific surgeries, significant differences between the pre- and postoperative scores on the CLEFT-Q HRQL scales (ie, psychological and social) were not observed. The HRQL construct represents a more distant construct than appearance in the context of cleft-related surgery outcomes, which may explain the change in HRQL scales being smaller than on the appearance scales.

Our study has several limitations. Importantly, we did not achieve the projected sample size of 50 participants per operation within the study timeframe. This limitation may have impacted the ability to estimate change in HRQL (ie, psychological and social) following cleft surgery. A large proportion of participants were lost to follow-up due to logistical reasons, such as not being able to reach them in clinic at postoperative appointments or via phone/email 6 months after the surgery. After three attempts to phone/email, the participant was considered lost to follow up. In addition to cleft-related surgery, factors such as age, gender, number of siblings and type of cleft lip and/or palate have shown to affect patients’ HRQL and therefore may have influenced the collected HRQL scale scores (Damiano et al., 2007; Kramer et al., 2008; Mani et al., 2010).

Further, HRQL improvements may require a longer period between assessments and a combination of treatment modalities provided by the entire healthcare team. While those lost to follow up were representative of the larger sample of participants, the number of participants lost to follow up and variation in response rates between countries limit this study's generalizability. Another limitation to the generalizability of this study was the lack of data collected on the ethnicity and family's socioeconomic status. Moreover, the information about the surgical approaches used at each center by each surgeon was not collected. The time between the pre- and postoperative assessments varied within each operation. Finally, participants were treated at different sites within and between countries. Differences in how patients with CL/P are managed internationally could have introduced variability to the postoperative scores between sites. Future responsiveness studies should aim to use standardized protocols. Finally, an inability to examine responsiveness and approximate MIDs for all CLEFT-Q scales was a limitation. Some scales were not relevant to specific surgeries, and some scales are appropriate for specific age-groups. Further research could examine responsiveness and determine MIDs for the scales that were not examined in this study.

## Conclusion

This study examined internal responsiveness of the CLEFT-Q using distribution-based approaches. Despite the small sample, we demonstrated that appearance scales were highly responsive to measuring change following cleft-specific surgeries. In addition to detecting statistically significant change on several appearance scales for each procedure, the largest change was found on the scale most closely associated with the procedure. Finally, MIDs determined using two distribution-based approaches provide a range to gauge whether the CLEFT-Q scale scores are clinically significant. Further examination of MIDs for patients with CL/P using the anchor-based approach in a larger and more clinically and culturally diverse sample is needed.

## Supplemental Material

sj-docx-1-cpc-10.1177_10556656211064479 - Supplemental material for A Prospective Study to Examine Responsiveness and Minimally Important Differences (MIDs) for the CLEFT-Q Scales Following Three Cleft-Specific Operations Supplemental material, sj-docx-1-cpc-10.1177_10556656211064479 for A Prospective Study to Examine Responsiveness and Minimally Important Differences (MIDs) for the CLEFT-Q Scales Following Three Cleft-Specific Operations by A Miroshnychenko, C Rae, K Wong Riff, CR Forrest, T Goodacre, MC Swan, R Slator, J Goldstein, A Thoma, K Harman and A Klassen in The Cleft Palate Craniofacial Journal

sj-docx-2-cpc-10.1177_10556656211064479 - Supplemental material for A Prospective Study to Examine Responsiveness and Minimally Important Differences (MIDs) for the CLEFT-Q Scales Following Three Cleft-Specific Operations Supplemental material, sj-docx-2-cpc-10.1177_10556656211064479 for A Prospective Study to Examine Responsiveness and Minimally Important Differences (MIDs) for the CLEFT-Q Scales Following Three Cleft-Specific Operations by A Miroshnychenko, C Rae, K Wong Riff, CR Forrest, T Goodacre, MC Swan, R Slator, J Goldstein, A Thoma, K Harman and A Klassen in The Cleft Palate Craniofacial Journal
