# Supplementary material for: A Prospective Study to Examine Responsiveness and Minimally Important Differences (MIDs) for the CLEFT-Q Scales Following Three Cleft-Specific Operations
Source: Cleft Palate Craniofac J. 2021 Dec 14;60(4):413–20. doi: 10.1177/10556656211064479 (PMC10018053; doi:10.1177/10556656211064479)
Supplement: sj-docx-1-cpc-10.1177_10556656211064479 - Supplemental material for A Prospective Study to Examine Responsiveness and Minimally Important Differences (MIDs) for the CLEFT-Q Scales Following Three Cleft-Specific Operations [file sj-docx-1-cpc-10.1177_10556656211064479.docx]

| Recruitment methodology before and after operation at each site. | | | | | |
| --- | --- | --- | --- | --- | --- |
| Site | **Recruiter** | **Location (preop)** | **Data collection** | **Location (postop)** | **Data collection** |
| The Hospital for Sick Children | Researcher | Hospital | Tablet | Home | Electronic |
| Children’s Hospital of Pittsburgh | Researcher | Clinic/home | Paper | Clinic/home | Paper |
| Broomfield Hospital | Research nurse | Clinic | Paper | Clinic | Paper |
| University Hospitals Birmingham | Research nurse | Clinic | Paper | Clinic | Paper |
| Great Ormond Street Hospital | Psychologist | Clinic | Paper | Clinic | Paper |
| Oxford & Salisbury Cleft Centers | Research nurse | Clinic | Paper | Clinic | Paper |

**Appendix 1**
