# Supplementary material for: A Prospective Study to Examine Responsiveness and Minimally Important Differences (MIDs) for the CLEFT-Q Scales Following Three Cleft-Specific Operations
Source: Cleft Palate Craniofac J. 2021 Dec 14;60(4):413–20. doi: 10.1177/10556656211064479 (PMC10018053; doi:10.1177/10556656211064479)
Supplement: sj-docx-2-cpc-10.1177_10556656211064479 - Supplemental material for A Prospective Study to Examine Responsiveness and Minimally Important Differences (MIDs) for the CLEFT-Q Scales Following Three Cleft-Specific Operations [file sj-docx-2-cpc-10.1177_10556656211064479.docx]

**Appendix 2**

| Formulas used in the distribution-based approach. | |
| --- | --- |
| Variable | **Formula** |
| Cohen’s d | $Cohen^{'}s d=(N1-N2)/SDpooled$  $SDpooled=$√(((SD_1_^2^+SD_2_^2^)/2))  Or  $Cohen^{'}s d=Mean/SDbaseline$ |
| Standardized Response Mean | $SRM=Mean/SDchange$ |
| MID ½ SD for Parametric Data | $MID=SDchange/2$ |
| MID ½ ES for Parametric Data | $MID= ½0.5\times SDbaseline$ |
